# Ecology and application of haloalkaliphilic anaerobic microbial communities

**DOI:** 10.1007/s00253-015-6937-y

**Published:** 2015-09-10

**Authors:** João A.B. Sousa, Dimitry Y. Sorokin, Martijn F.M. Bijmans, Caroline M. Plugge, Alfons J.M. Stams

**Affiliations:** 1 Laboratory of Microbiology, Wageningen University, Dreijenplein 10, 6703 HB Wageningen, Netherlands; 2 Wetsus, European Centre of Excellence for Sustainable Water Technology, Oostergoweg 9, 8911 MA Leeuwarden, Netherlands; 3 Winogradsky Institute of Microbiology, Research Centre of Biotechnology, Russian Academy of Sciences, Moscow, Russia; 4 Department of Biotechnology, Delft University of Technology, 2628 BC Delft, Netherlands; 5 Department of Biological Engineering, University of Minho, Braga, Portugal

**Keywords:** Anaerobic, Haloalkaline, Haloalkaliphilic, Fermentation, Lignocellulosic feedstocks, Methane, Sulfidogenesis, Toxicity

## Abstract

Haloalkaliphilic microorganisms that grow optimally at high-pH and high-salinity conditions can be found in natural environments such as soda lakes. These globally spread lakes harbour interesting anaerobic microorganisms that have the potential of being applied in existing technologies or create new opportunities. In this review, we discuss the potential application of haloalkaliphilic anaerobic microbial communities in the fermentation of lignocellulosic feedstocks material subjected to an alkaline pre-treatment, methane production and sulfur removal technology. Also, the general advantages of operation at haloalkaline conditions, such as low volatile fatty acid and sulfide toxicity, are addressed. Finally, an outlook into the main challenges like ammonia toxicity and lack of aggregation is provided.

## Introduction

The metabolic potential of anaerobic microorganisms has been exploited in a wide range of applications, like volatile fatty acids (VFAs), alcohols, H_2_ and methane production. However, information about the application of haloalkaliphilic anaerobes that thrive in high-pH (>8.5) and high-salt conditions (>35 g l^−1^) is very limited.

In these extreme environments, microorganisms adapted physiological mechanisms to cope with high pH and salinity. The high salinity of the environment must be compensated to prevent osmotic stress and water leakage from the cell. To cope with high salinity, microorganisms accumulate inorganic or organic compounds that work as osmoregulators, preventing the loss of water inside the cell (Dektova and Boltyanska 2007). The high pH, on the other hand, affects the proton balance and transport by the ATPases that are responsible for ATP production. Even though the pH of the environment is alkaline, the cell inside usually operates close to neutral pH. Cells cope with this by having more negatively charged cell walls that generate a layer of more concentrated protons, lower pH, near the cell while repelling anions. These adaptations to alkaline conditions have already been recently reviewed in more detail (Banciu and Muntyan 2015; Preiss et al. 2015).

Various haloalkaline environments, like soda lakes, soda solonchak soil, mining industry waste and leafs of salt secreting trees, have been described (Qvit-Raz et al. 2008; Sorokin et al. 2008; Sorokin et al. 2014; 2015a; Santini et al. 2015). However, only soda lakes and soda solonchak soils have the buffer capacity to maintain a high pH (> 8.5) and high salinity (> 35 g l^−1^). Soda solonchak soils have a higher aeration when compared to soda lakes and favour aerotolerant microorganisms (Sorokin et al. 2008). Thus, soda lakes are the most suitable habitats to find anaerobic haloalkaliphilic microorganisms. In these lakes, a high pH and salinity is caused by the evaporative concentration of soluble sodium carbonates as a result of low concentrations of divalent cations such as calcium or magnesium in the ground waters and surrounding minerals. The extremely high pH (between 9 and 11) is stable due to a high alkaline buffering capacity of soluble carbonates and salinity can go from 35 g l^−1^ up to saturation. Soda lakes harbour highly active and diverse microbial communities involved in the carbon, sulfur and nitrogen cycles. Microbiological studies on soda lakes have been reviewed by Sorokin et al. (2014; 2015a) Also, reviews on application of haloalkaliphilic microorganisms on nitrogen cycle, sulfide oxidation, heavy metals removal, biofuel production and enzyme production are available (Horikoshi 1999; Zhao et al 2014).

In this mini-review, research focused on potential application of anaerobic haloalkaliphilic microorganisms in fermentation of lignocellulosic feedstocks, methane production and sulfur removal technology will be reviewed. The advantages of low VFAs and sulfide toxicity and high methane content will be discussed. We will also focus on the main technological challenges, such as ammonia toxicity and lack of microbial aggregation.

## Anaerobic digestion of lignocellulosic feedstocks

The rate of hydrolysis of sugar polymers is crucial in the fermentation of lignocellulosic feedstocks by anaerobic fermentative bacteria. These feedstocks include waste from agriculture, forest and paper industry where the hydrolytic step is a bottleneck. This is mainly due to the highly packed crystal structure of the fibres composed of lignin, cellulose and hemicellulose (Mathews et al. 2015). To improve hydrolysis, an alkaline pre-treatment can be performed to reduce the fibre crystallinity, making them more accessible to attack of microbial hydrolases (Hendriks and Zeeman 2009).

## Fermentation

After alkaline pre-treatment, the current approach is biofermentation at neutral pH after neutralizing the alkaline broth. However, the use of haloalkaliphilic microorganisms eliminates the need for pH adjustments, thus reducing costs (Porsch et al. 2015). The information on haloalkaliphilic cellulolytic anaerobes is, so far, limited to a few soda lake alkaliphiles. *Clostridium alkalicellulosi* (Table 1) (Zhilina et al. 2005; Zvereva et al. 2006) is able to produce acetate, ethanol, lactate, hydrogen and traces of formate as products during fermentation of cellulose and cellobiose. Pikuta et al (2006) reported that *Anaerovirgula multivorans* can weakly grow on cellulose in alkaline medium supplemented with yeast extract. However, no growth kinetics and activity data have been provided. The sugars released from the lignocellulosic feedstocks during alkaline pre-treatment can be used by many cultured haloalkaliphilic saccharolytic fermenters. Such bacteria, belonging to the genera *Spirochaeta*,*Amphibacillus*,*Alkaliflexus* and *Alkalitalea*, were isolated from different soda lakes and are capable of fermenting cellobiose and glucose, the main product of cellulose hydrolysis (Table 1) (Zhilina et al. 2001; Zhilina et al. 2004; Pikuta et al. 2009; Zhao and Chen 2012). The fermentation products varied between species but are mainly acetate, ethanol, lactate and hydrogen. However, *Halanaerobium hydrogeniformans* produced acetate, formate and hydrogen as main products in a haloalkaline fed-batch bioreactor fed with hydrolysed switchgrass (Table 1) (Begemann et al. 2012). Ethanol, lactate and hydrogen can be used by haloalkaliphilic acetogens, such as *Natroniella* and *Fuchsiella*, converting them to acetate (Table 1) (Zhilina et al. 2012).

**Table 1:** Relevant haloalkaliphilic microorganisms for fermentation of lignocellulosic feedstocks at haloalkaline conditions and their role, optimum pH and optimum salinity

Microorganism	Metabolic type	Optimum pH	Optimum salinity (M Na^+^)	Reference
*Clostridium alkalicellum*	Cellulolytic/fermenter	9	0.15–0.3	Zhilina et al. 2005
*Anaerovirgula multivorans*	Cellulolytic^a^/fermenter	8.5	0.17–0.34	Pikuta et al. 2006
*Spirochaeta alkalica*	Fermenter	8.7–9.6	0.5–1.7	Pikuta et al. 2009
*Spirochaeta aficana*	Fermenter	8.8–9.75	0.85–1.2	Pikuta et al. 2009
*Spirochaeta asiatica*	Fermenter	8.4–9.4	0.5–1	Pikuta et al. 2009
*Amphibacillus tropicus*	Fermenter	9.5–9.7	0.17–3.6	Zhilina et al. 2001
*Amphibacillus fermentum*	Fermenter	8–9.5	1.87	Zhilina et al. 2001
*Alkaliflexus imshenetskii*	Fermenter	8.5	0.35	Zhilina et al. 2004
*Alkalitalea saponilacus*	Fermenter	9.7	0.44–0.69	Zhao and Chen 2012
*Halanaerobium hydrogeniformans*	Fermenter	11	1.3	Begemann et al. 2012
*Natroniella acetigena*	Acetogen	9.7–10	2.1–2.7	Zhilina et al. 2012
*Fuchsiella alkaliacetigena*	Acetogen	8.8–9.3	2.8–3.3	Zhilina et al. 2012

^a^More information is required to clearly prove that *Anaerovirgula multivorans* is capable of growing on cellulose

## Methane production

**Fig. 1:**
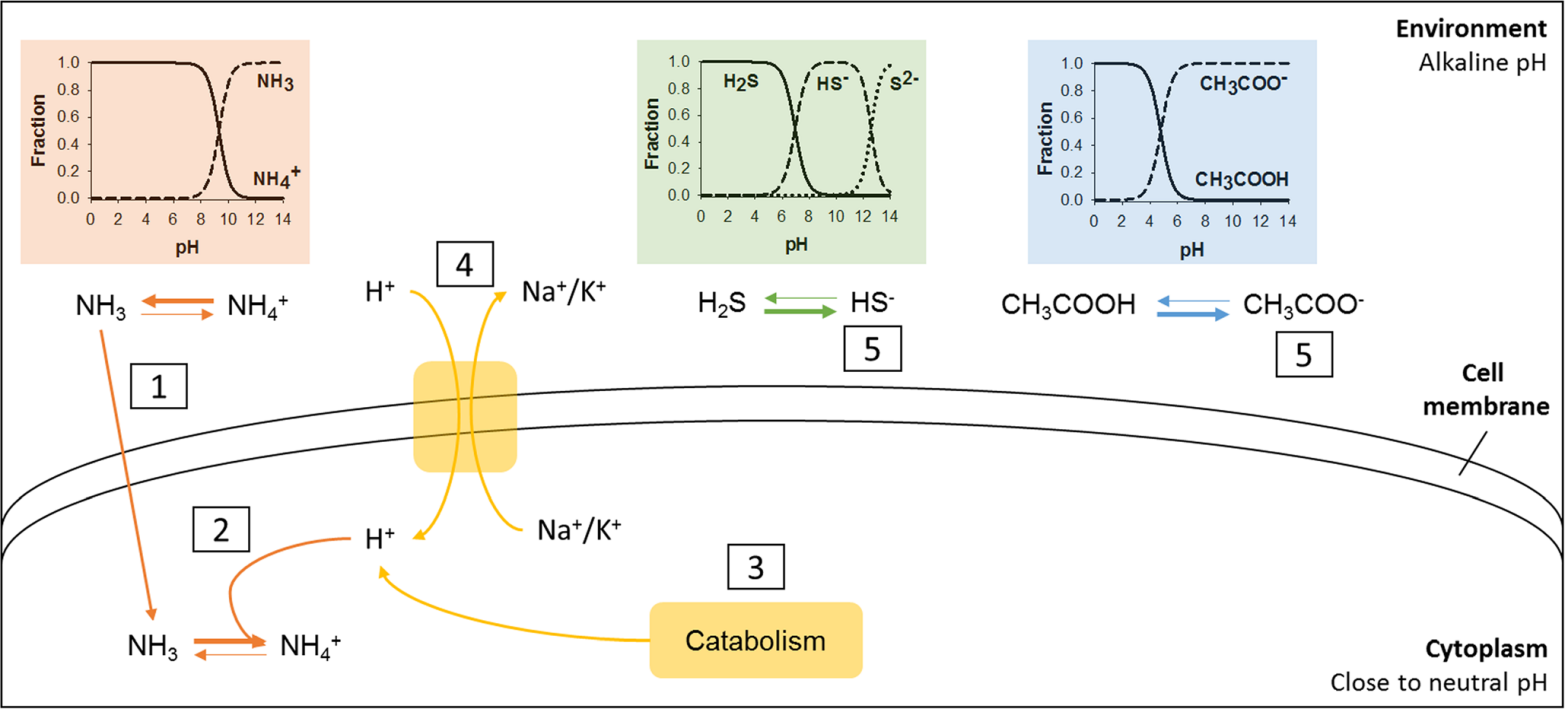
Effect of ammonia, sulfide and acetate (representing VFAs in general) on microorganisms living at alkaline pH and chemical equilibrium of ammonia sulfide and acetate at different pH values. *1*—at alkaline pH, ammonia tends to the un-ionized species (NH_3_) which can cross cell membranes in contrast with the ionized species (NH_4_^+^); *2*—due to the close to neutral pH inside the cell, the chemical equilibrium shifts towards the NH_4_^+^ species, consuming one proton (H^+^) and disrupting the proton balance; *3*—to compensate the lost H^+^, the primary source of H^+^ is from the catabolic reactions; *4*—also, antiporters in the cell membrane may pump H^+^ in and simultaneously pump sodium (Na^+^) or potassium (K^+^) out, generating an osmotic difference that needs to be compensated; *5*—at alkaline pH, sulfide and acetate exist in the ionized form, HS^−^ and CH_3_COO^−^, which cannot easily pass the cell membrane

Methanogenic fermentation of wastes at haloalkaline conditions can be an interesting option for renewable biogas production. At high pH, VFA toxicity is reduced because VFA are mostly present in the dissociated form which cannot easily cross cell membranes and disrupt the proton balance (Fig. 1). This would allow the operation of such bioreactors at higher organic loadings. At high pH, the CO_2_ is more retained as carbonates which could lead to a lower CO_2_ content in the biogas. Also, sulfide at high pH is mainly in the ionized form (HS^−^) which is less volatile and toxic, resulting in a gas with very low concentrations of sulfide. A recent study on the digestion of the microalgae *Spirulina* at haloalkaline conditions resulted in a biogas with a methane content of 96 % and without traces of sulfide (Nolla-Ardèvol et al 2015). This might reduce the need for biogas post-treatment to remove CO_2_ and H_2_S, allowing the use of the biogas directly in natural gas supply grid.

In soda lakes, the methanogenic activity in the sediments is similar to freshwater lakes and marine sediments (Kuivila et al. 1989;1990; Sorokin et al. 2015b). Just a few methanogenic archaea have been isolated from soda lakes (Zhilina et al. 2013; Sorokin et al. 2015b). The isolated hydrogenotrophic methanogens belong to the genus *Methanocalculus* that use H_2_ and formate. The isolated methylotrophic methanogens belong to the genera *Methanolobus* and *Methanosalsum* that use various methylated compounds. Acetate conversion to methane is also possible, albeit at very low rates, either directly at moderate salinity by alkaliphilic *Methanosaeta* or by syntrophic associations of reversed acetogens and lithotrophic *Methanocalculus* at moderate to high salinity (Sorokin et al., 2015b).

## Sulfidogenesis

Bioreduction of inorganic sulfur compounds can be applied to treat sulfur-rich waste streams with high pH and salinity originated from the oil, natural gas and mining industries. When such streams are exposed to oxygen, mainly oxidized compounds exist like sulfate, thiosulfate, sulfite or sulfur. However in the environment, such compounds might be reduced, producing sulfide which is toxic and characterized by the rotten egg smell. To prevent this, the sulfur compounds can be removed from such streams by microbial processes. Oxidized sulfur compounds can be reduced to sulfide in a controlled environment, like a bioreactor. The sulfide produced can be biologically oxidized at oxygen-limited conditions to elemental sulfur, which has economic value (Janssen et al. 2009). Sulfur cycle-related haloalkaliphilic bacteria can be found in soda lakes where the microbial sulfur cycle is very active (Sorokin et al. 2011). Haloalkaliphilic sulfate-reducing bacteria (SRB) that use besides sulfate also thiosulfate and sulfite as electron acceptor and often can disproportionate thiosulfate and sulfite have also been isolated from soda lakes. Both lithotrophic and organotrophic SRB have been described and reviewed by Sorokin et al (2011; 2014; 2015a). Elemental sulfur was never shown to be reduced by haloalkaliphilic SRB. However, the specialized sulfur-reducing bacteria, which can reduce or disproportionate elemental sulfur, are also present in these environments (Sorokin et al. 2014; 2015a).

Sulfate reduction at haloalkaline conditions was tested in anaerobic filters and gas lift bioreactors using various electron donors (Zhou and Xing 2015; Sousa et al. 2015). These results revealed that the most reliable electron donors are formate, hydrogen and ethanol (Table 2). The dominant SRB found in these bioreactors belonged to the lithotrophic genera *Desulfonatronospira* and *Desulfonatronovibrio*. As produced sulfide at high pH is present in the dissociated form (HS^−^), this has a much lower toxicity to the biomass compared to neutral pH (Fig. 1). Sousa et al (2015) showed that sulfate reduction occurred up to 260 mM of sulfide at pH 9, while at pH 7 the sulfide toxicity was already severe at 30 mM (Van Houten et al. 1994). Therefore, bioreactors operated at haloalkaline conditions can handle more concentrated sulfur streams than at neutral pH.

**Table 2:** Comparison of different studies on sulfate reduction in bioreactors operated at haloalkaline conditions

Reference	Sousa et al. 2015	Zhou and Xing 2015	Zhou and Xing 2015
Reactor type	Gas lift with three-phase separator	Anaerobic filter	Anaerobic filter
e^−^ acceptor	Sulfate	Sulfate	Sulfate
e^−^ donor	H_2_	Formate	Ethanol
pH	9	9.5	9.5
Na^+^ conc. (M)	1.5	1	1
Temperature (°C)	35	37	37
HRT (day)	3.3	1	1
Conversion rates (mmol l^−1^ day^−1^)	18	85	89.5
Max. sulfide conc. (mmol l^−1^)	260	76	82
Side products	Formate	Acetate	Acetate/formate/lactate
Biomass conc. (mg l^−1^)	7.2 (±3)	N.D.	N.D.
Biomass aggregation	No aggregation	N.D.	N.D.

*N.D.* not described

## Future challenges for haloalkaline bioreactor research

The application of anaerobic haloalkaline microbial communities has numerous advantages. But even though these microorganisms are highly adapted to these extreme conditions, there are challenges to overcome before applying such technologies at full scale.

### Ammonia toxicity

One challenge is the ammonia toxicity at high pH (Fig. 1). At haloalkaline conditions un-ionized ammonia (NH_3_) rather than ammonium (NH_4_^+^) is the dominant chemical species, as the pKa is 9.25. NH_3_ can freely diffuse through the cell membrane and disrupt the proton balance inside the cells, making it toxic while NH_4_^+^ cannot cross the membrane and, therefore, is not toxic (Kayhanian 1999). After crossing the membrane, NH_3_ is protonized into NH_4_^+^ due to the near neutral pH in the cytoplasm maintained by alkaliphiles which, in turn, may weaken its neutral buffering. To compensate this, haloalkaliphiles primarily use protons from the catabolic reactions or can also use antiporters to transport protons into the cell while transporting potassium or sodium out of the cells (Kayhanian 1999). This use of antiporters, however, would generate additional osmotic stress that needs to be compensated.

### Lack of aggregation

The high pH and salinity in bioreactors can prevent a stable aggregation of microorganisms which is usually essential for biomass retention of slowly growing organisms. Previous studies showed that aggregation in bioreactors at high pH and salt concentrations did not occur at all or that stable granules disintegrated in high salinity bioreactors (Ismail et al. 2008; Sousa et al. 2015). The causes for this are still in discussion, and different mechanisms are proposed. At high pH, the hydrophobicity of cell surfaces and extracellular polymeric substance (EPS) might change and hydrophobicity has been reported to affect the microbial attachment (van Loosdrecht et al. 1987; Otto et al. 1999). Another possibility was proposed by Ismail et al (2008) who suggested that at high Na^+^ concentrations, Na^+^ replaces divalent cations, such as Ca^2+^, in the EPS matrix of aggregates, making the aggregates less stable. Another possible effect could be downregulation of carbon metabolism at high salinities as reported by He et al (2010). This subsequently lowers the EPS production in favour of osmolites production to balance the high salinity. Yet, halophilic isolates from the *Halomonas* genus were shown to produce EPS, and this could point to a significant role of high pH in the lack of aggregation at haloalkaline conditions (Martínez-Cánovas et al. 2004). To overcome the challenge of no aggregation at haloalkaline conditions, technologies like use of a biofilm support material in the reactor or a membrane biological reactor (MBR) should be considered.

### Operational challenges

Additional factors related to the engineering of haloalkaline bioreactors need to be addressed. By operating bioreactors at high salt and high pH, there is an increased risk of scaling if divalent cations are added. This problem requires special attention when designing and optimizing the processes. Also, the high pH and salinity effluent might require additional treatment to neutralize pH and salinity prior to its discharge.

## Conclusions and future prospects

Application of haloalkaliphilic anaerobic microbial communities in the abovementioned processes is an interesting route to consider in specific cases and/or to increase their efficiency. Operation at haloalkaline conditions has several advantages, like low VFA and sulfide toxicity, production of low CO_2_-containing and H_2_S-containing biogas and reduced need for pH control. On the other hand, the challenges of ammonia toxicity and lack of biomass aggregation need to be overcome for application in an industry. In general, more laboratory-scale bioreactor studies focusing on these microorganisms are required. Information on reaction rates, biomass growth and microbial communities during long-term experiments in bioreactors is essential to scale up these technologies.

### Aknowledgments

This work was performed in the TTIW-cooperation framework of Wetsus, European Centre of Excellence for Sustainable Water Technology (www.wetsus.nl). Wetsus is funded by the Dutch Ministry of Economic Affairs, the European Union Regional Development Fund, the Province of Fryslân, the City of Leeuwarden and the EZ/Kompas program of the “Samenwerkingsverband Noord-Nederland”. The authors would like to thank the participants of the research theme “Sulfur”, namely Paqell, for fruitful discussions and financial support.
